# An Interpretable Multimodal Machine-Learning Model for Predicting Cognitive Impairment from Routine MRI: A Study with External Validation

**DOI:** 10.3390/bioengineering13101107

**Published:** 2026-09-23

**Authors:** Xiu Chen, Baowen Zhang, Mengdie Li, Song Wang, Mei Yang, Jiejing Zhang, Xuying Zhu, Yingjie Kang, Wenli Tan

**Affiliations:** 1 Department of Radiology, Shuguang Hospital Affiliated with Shanghai University of Traditional Chinese Medicine, 528 Zhangheng Road, Shanghai 201203, China; cx1030950937@gmail.com (X.C.); zhangbaowen423@163.com (B.Z.); silenceli326@163.com (M.L.); 15155919083@163.com (M.Y.); qcandzrq@163.com (J.Z.); 2 Department of Radiology, Longhua Hospital Affiliated with Shanghai University of Traditional Chinese Medicine, 725 Wanping South Road, Shanghai 200032, China; songwangws@163.com; 3 Department of Neurology, Shuguang Hospital Affiliated with Shanghai University of Traditional Chinese Medicine, 528 Zhangheng Road, Shanghai 201203, China; 13651800066@163.com

**Keywords:** cognitive impairment, magnetic resonance imaging, multimodal machine learning, SHAP, white matter hyperintensities

## Abstract

Existing MRI-based prediction models for cognitive impairment often rely on a single biomarker or simply compare algorithms, act as black boxes, and lack external validation, which limits their clinical translation. We developed and externally validated an interpretable multimodal machine-learning model that classifies a MoCA-defined screening outcome as a binary task: cognitive impairment (CI, MoCA < 26) versus no cognitive impairment (NCI, MoCA ≥ 26). Sixteen candidate features were considered, including AI-derived medial temporal atrophy (MTA), regional white matter hyperintensity (WMH) volumes and proportions, and Fazekas ratings from routine T1WI and T2-FLAIR, together with age and sex. In a single-center cohort of 433 subjects (302 CI, 131 NCI) with a 7:3 training/hold-out split, five algorithms were compared and Random Forest performed best; SHAP-guided recursive feature elimination within five-fold cross-validation then reduced the set from 16 to 11 features. The optimized 11-feature Random Forest reached a cross-validated AUC of 0.883 ± 0.024 on the training folds and an internal hold-out AUC of 0.966, and 0.860 in an independent ADNI cohort of 139 subjects (100 CI, 39 NCI). SHAP identified the left MTA score as the most influential feature, followed by near-ventricular (juxtaventricular and periventricular) WMH burden and the right MTA score. The left MTA score was most strongly correlated with MoCA (r = −0.481). The model may offer an objective, routine-MRI-based screening aid, pending prospective multicenter validation.

## 1. Introduction

The incidence of cognitive impairment is rising markedly as the population ages. Early identification of cognitive impairment is critical for implementing effective interventions, improving patients’ quality of life, and reducing the socioeconomic burden [1,2]. At present, the diagnosis of cognitive impairment relies primarily on neuropsychological assessments. However, these assessments can be limited by subjectivity, variable standardization, and relatively low sensitivity in the early stages [3]. Recent work on fluid biomarkers [4] and imaging-based AI screening models has improved early detection [5,6]. Even so, few existing approaches are both objective and low-cost enough for routine clinical use. A standardized and accessible screening tool is therefore still needed.

Imaging offers a practical route to such a tool, but its clinical value depends on the technique. Functional MRI techniques such as blood-oxygen-level-dependent functional MRI (BOLD-fMRI) and diffusion tensor imaging (DTI) have been widely used in cognitive impairment research. However, they often require long scan times and complex postprocessing [7], which limits their use for individual patients and for rapid screening. By contrast, brain atrophy and white matter hyperintensity (WMH) are also closely associated with cognitive impairment [8,9] and can be assessed far more simply. Standardized visual tools such as the medial temporal lobe atrophy (MTA) score and Fazekas rating are already used in diagnosis [10,11]. They can be obtained from 3D-T1WI and T2-FLAIR in only 1–3 min and need no complex postprocessing. This makes them practical in routine practice. Yet these visual assessments remain subjective and cannot integrate information across multiple features into a single judgment.

Artificial intelligence has improved MRI analysis [12,13] and is increasingly applied to cerebral small vessel disease and cognitive impairment [14]. Automated segmentation can identify and quantify imaging biomarkers such as WMH and brain atrophy from MR images [15,16]. These quantitative phenotypes are objective and reduce the subjectivity of visual rating [17]. Using such features, earlier machine-learning studies have distinguished mild cognitive impairment from cognitively normal individuals [5,6]. Nevertheless, several challenges remain. Many models rely on a single biomarker or focus on comparing algorithms [18] rather than integrating complementary features [19]. Many also act as black boxes, and their limited interpretability may reduce clinical trust [20], which has increased interest in explainable-AI methods [21]. External validation across populations and scanners is likewise scarce, so generalizability remains uncertain [22].

To address these gaps, we developed an interpretable multimodal model to predict MoCA-defined cognitive impairment. Rather than relying on a single biomarker, the model integrates AI-derived measures of medial temporal atrophy (a neurodegenerative marker) and white matter hyperintensity (a vascular marker) with the clinical variables age and sex. These imaging biomarkers require only routinely acquired T1WI and T2-FLAIR sequences. SHAP analysis is then used to interpret each predictor’s contribution to the model’s predictions, and the model is externally validated on the Alzheimer’s Disease Neuroimaging Initiative (ADNI) dataset. Our aim was to evaluate whether such an interpretable multimodal model could support the early screening of cognitive impairment in routine clinical practice.

## 2. Materials and Methods

### 2.1. Subjects

This was a single-center, retrospective, cross-sectional study, with external validation on an independent multicenter cohort (the ADNI dataset). The study was approved by the Ethics Committee of Shuguang Hospital Affiliated with Shanghai University of Traditional Chinese Medicine (No. 2022-1218-155-01), and all subjects gave written informed consent. All subjects completed the Montreal Cognitive Assessment (MoCA) and MRI, and were classified as cognitively impaired (CI, MoCA < 26) or not (NCI, MoCA ≥ 26) [23].

The inclusion criteria were: (1) aged 50 years or older for either sex; (2) right-handed. The exclusion criteria were: (1) MR contraindications; (2) the presence of neurological disorders, such as brain tumors, large cerebral infarction or hemorrhage, or obvious brain parenchymal defects; (3) significant head-motion artifacts on MR images. The internal cohort comprised 433 subjects (302 CI, 131 NCI).

For external validation, an independent cohort was drawn from the ADNI 3 phase (URL (https://adni.loni.usc.edu, accessed on 5 December 2025): participants scanned between January 2018 and October 2022 with 3D-T1WI, T2-FLAIR and a concurrent MoCA were selected by the same inclusion/exclusion criteria and MoCA < 26 threshold as the internal cohort, excluding those with missing data or images unsuitable for automated feature extraction. This yielded 139 subjects (100 CI, 39 NCI).

### 2.2. MR Data Acquisition and Image Feature Extraction

MR data acquisition: Internal subjects were scanned on a 3.0-T system (uMR 780, United Imaging Healthcare, Shanghai, China) with a 32-channel head coil. 3D T1-weighted imaging (3D-T1WI) was acquired with TR 7.24 ms, TE 3.1 ms, flip angle 10°, and 1 × 1 × 1 mm^3^ voxels, as well as T2-FLAIR with TR 8000 ms, TE 127.8 ms, TI 2420 ms, flip angle 90°, and 1 × 0.85 × 5 mm^3^ voxels. For the external cohort, images were obtained at multiple ADNI sites on 3-T scanners under ADNI-standardized protocols (adni.loni.usc.edu).

MR image feature extraction. Imaging features were extracted with the uAI Research Portal (Shanghai United Imaging Intelligence Co., Ltd., Shanghai, China; version 1.0) [24], which automatically segments brain structures and quantifies white matter lesions. Sixteen candidate features were derived. Medial temporal lobe atrophy (MTA) was scored separately for the left and right hemisphere (ordinal scale, 0–4). White matter hyperintensity (WMH) burden was quantified in four regions, defined by distance from the ventricular surface or the cortical surface: juxtaventricular (≤3 mm from the ventricle), periventricular (3–13 mm from the ventricle), deep (between the periventricular and juxtacortical regions), and juxtacortical (≤4 mm from the gray–white matter junction). For each region, both absolute volume (mL) and the proportion of total WMH volume (%) were recorded. The software also generated periventricular and deep Fazekas scores; the total Fazekas score was calculated as their sum. A software-derived Stroke score (1–5) indexed overall cerebrovascular lesion burden (Figure 1). Age and sex were included as clinical variables. MTA, Fazekas, and Stroke scores were ordinal outputs of the automated pipeline; no feature required manual rating. Images from the external ADNI cohort were processed with the same software and the same procedure.

### 2.3. Data Balancing and Model Development

The prediction task was binary classification of cognitive impairment (CI) versus no cognitive impairment (NCI). Internal cohort groups differed in size (302 CI, 131 NCI), so the synthetic minority oversampling technique (SMOTE, k = 5) was applied to the internal dataset to generate additional NCI samples before any data partitioning [25,26,27]. The external cohort was not oversampled.

After oversampling, the internal dataset was randomly divided into a training set and a hold-out set in a 7:3 ratio with a fixed random seed. All models were fitted on the training set. The hold-out set was then used to compare the five candidate algorithms and to determine the number of features retained in the final model.

The five algorithms were Random Forest (RF) [28], support vector machine (SVM), multilayer perceptron neural network (NN), logistic regression (LR), and XGBoost. All models were implemented in Python 3.8 (scikit-learn 1.0.2; imbalanced-learn 0.9.0; SHAP 0.41.0) using fixed configurations without hyperparameter search. Class weighting (class_weight = ‘balanced’) was enabled for RF, SVM, and LR. Complete model settings are provided in the Supplementary Material.

### 2.4. Model Performance Evaluation and Interpretability Analysis

The five algorithms were compared on the internal hold-out set. Performance was summarized by the area under the receiver operating characteristic curve (AUC) and by confusion-matrix metrics: accuracy, precision, recall, and F1 score, where TP, TN, FP, and FN denote true positives, true negatives, false positives, and false negatives. The positive class was cognitive impairment. The algorithm with the highest AUC was selected for subsequent feature optimization and interpretability analysis.

SHAP (SHapley Additive exPlanations), rooted in cooperative game theory, was used to quantify each feature’s global and local contribution to the model output [29]. Starting from the algorithm with the highest hold-out AUC, SHAP-guided recursive feature elimination (RFE) was performed with five-fold stratified cross-validation on the training set [30,31], preserving the class distribution across folds. For every feature subset, both the cross-validated AUC and the hold-out AUC were recorded, and the number of features was chosen according to the hold-out AUC. The optimized model was then fitted on the 11 retained features using the same fixed configuration as in the algorithm comparison (a andom orest with n_estimators = 100 and class_weight = ‘balanced’, the remaining hyperparameters left at their scikit-learn defaults; full settings in the Supplementary Material); no hyperparameters were tuned on the hold-out set.

In the interpretability analysis, SHAP values refer to the model’s predicted probability of cognitive impairment: positive values shift the prediction toward CI, and negative values toward NCI. Spearman rank correlations among features were computed to assess multicollinearity.

### 2.5. Statistical Analysis

All statistical analyses were performed using SPSS (version 25.0; IBM Corp., Armonk, NY, USA) and Python (version 3.8). Continuous variables were first assessed for normality using the Shapiro–Wilk test. Normally distributed data were expressed as mean ± standard deviation, whereas non-normally distributed data were reported as median and interquartile range. Between-group comparisons of categorical variables were conducted using the chi-square test. For continuous variables, the two-tailed independent-samples *t* test was applied when normality assumptions were met, while the Mann–Whitney U test was used for non-normally distributed data. Correlations between variables were evaluated using Spearman’s rank correlation coefficient. The 95% confidence intervals for the AUC were estimated by bootstrapping with 1000 resamples. All statistical tests were two-sided, and a *p* value < 0.05 was considered statistically significant.

## 3. Results

### 3.1. Clinical Characteristics

The workflow of this study is shown in Figure 2. A total of 433 subjects were included: 302 with cognitive impairment (69.7%) and 131 without (30.3%) (Table 1). All subjects completed MRI examinations and imaging feature extraction, and the final dataset of 16 variables had no missing values.

### 3.2. Comparison of Machine Learning Algorithms

Among the five algorithms, Random Forest achieved the highest AUC on the internal hold-out set (0.941, 95% CI 0.903–0.979) and performed consistently across all metrics, whereas the neural network and XGBoost were competitive only on individual metrics (Figure 3). Random Forest was therefore selected for feature optimization and interpretability analysis.

### 3.3. Feature Selection and Model Performance

Based on SHAP feature importance, recursive feature elimination (RFE) reduced the 16 candidate features to 11 (Figure 4). The removed features were sex, the juxtaventricular WMH proportion, both juxtacortical WMH features, and the Stroke score. The cross-validated AUC was stable across the larger feature subsets: the 11-feature model gave 0.883 ± 0.024 (coefficient of variation 2.7%), close to 0.895 ± 0.021 for the full 16-feature model (Supplementary Table S1). Among all subsets, the 11-feature model gave the highest hold-out AUC (0.966, 95% CI 0.930–0.992), with accuracy 89.3%, precision 89.8%, recall 88.3% and F1 89.1% (Figure 5). In Figure 5A the shaded band denotes ±SD across folds.

### 3.4. External Validation

In the external ADNI cohort (n = 139; 100 CI, 39 NCI), the optimized 11-feature model achieved an AUC of 0.860 (95% CI 0.789–0.924) (Figure 6). At the operating threshold fixed in the internal cohort, sensitivity was 0.870, specificity was 0.513, PPV was 0.821, NPV was 0.606, and F1 was 0.845 (Supplementary Table S3).

Compared with the internal cohort, the ADNI cohort was older and included fewer women, whereas the overall MoCA distribution and cognitive impairment prevalence were similar (Table 2; Supplementary Table S2).

### 3.5. Model Interpretation

SHAP analysis identified the left MTA score as the most influential feature in the fitted model, with near-ventricular (juxtaventricular and periventricular) WMH burden and the right MTA score also among the leading contributors (Figure 7). However, because the WMH features were strongly intercorrelated (Figure 8, up to r ≈ 0.99), the importance of individual regions could not be uniquely attributed. The two MTA scores were strongly correlated with each other (r = 0.77). In contrast, atrophy and WMH features were only moderately correlated (r ≈ 0.3–0.6), and age showed weak-to-moderate correlations with the imaging features (r ≈ 0.2–0.4). All imaging features were negatively correlated with MoCA score, with the left MTA score (r = −0.48) and periventricular WMH vol% (r ≈ −0.46) being the strongest.

## 4. Discussion

In this study, we developed an interpretable multimodal machine-learning model for cognitive impairment that combines AI-derived MRI biomarkers with clinical variables and uses SHAP for interpretability. Random Forest performed best among the five algorithms. After SHAP-guided RFE, the optimized 11-feature model reached a cross-validated AUC of 0.883 in the internal cohort and 0.860 in an independent external cohort. SHAP highlighted the left MTA score as the most influential feature in the fitted model, with near-ventricular (juxtaventricular and periventricular) WMH burden and the right MTA score among the leading contributors.

Automated segmentation can derive quantitative imaging biomarkers from routine T1WI and T2-FLAIR, a more feasible workflow than advanced techniques such as DTI and BOLD-fMRI [32,33]. MTA and regional WMH volumes have long been associated with cognitive impairment in Alzheimer’s disease (AD) and vascular cognitive impairment (VCI) [9,34]. However, most prior studies validated a single biomarker or focused on algorithm comparison rather than integrating complementary markers [35]. This work demonstrates that neurodegenerative atrophy and vascular white matter injury from these routine sequences can be combined in a single interpretable framework that retains useful discrimination in an independent external cohort.

After selecting Random Forest among five algorithms, SHAP-guided RFE reduced the feature set from 16 to 11 with little change in cross-validated AUC. External discrimination was lower than internal performance (external AUC 0.860), as is common when a model is applied to a different population and imaging protocol. In terms of disease spectrum, the ADNI cohort showed greater medial temporal atrophy, whereas our cohort had a higher WMH burden. The near-absence of deep WMH in ADNI (median 0.06 vs. 1.62 mL) is unlikely to be purely biological and probably also reflects differences in FLAIR acquisition and automated segmentation. Images were further acquired at multiple ADNI centers and scanners, which may also contribute to the lower external AUC. External specificity was modest (0.513) at the operating threshold fixed in the internal cohort, indicating the over-classification of cognitive impairment in ADNI. The threshold would therefore need recalibration in the target population before screening use.

Medial temporal atrophy, especially on the left, was the most influential feature in the fitted model (Figure 4) and showed the strongest correlation with MoCA score (r = −0.48, Figure 8), consistent with the recognized role of hippocampal atrophy in cognitive impairment [9,36]. The left-sided dominance may reflect the asymmetric pathology of AD [37]. Near-ventricular (juxtaventricular and periventricular) WMH burden ranked relatively highly (Figure 7), a pattern that is biologically plausible. Periventricular lesions lie where major white matter tracts converge and have repeatedly been linked to cognitive impairment [38], and in our cohort periventricular WMH vol% showed the strongest correlation with MoCA (r ≈ −0.46), perhaps reflecting interruption of cognitive-network tracts or cerebral small vessel disease [34,39]. However, the WMH features were highly correlated, especially between the volume and proportional measures of the same WMH region (up to r ≈ 0.99), and SHAP distributed attribution among these near-duplicate predictors. We therefore regard near-ventricular WMH burden as a notable contributor without claiming the independent superiority of any single region. Within the model, AI-derived WMH volumes contributed more than visual Fazekas ratings (Figure 4). Nonetheless, SHAP importance reflects a feature’s role in the fitted model rather than comparative diagnostic accuracy, so this does not establish that quantitative measures are diagnostically superior. Atrophy and WMH correlated only moderately (r ≈ 0.3–0.6) and may capture complementary information along the neurodegenerative and vascular axes of the disease [40].

This study has several limitations. Cognitive status was defined by the MoCA alone, a screening rather than a diagnostic tool, so the outcome is a screening classification that a fuller neuropsychological testing would define more definitively. Only structural MRI was used, without metabolic or molecular markers (e.g., FDG-PET, APOE ε4, p-tau217, Aβ42) that might improve prediction [41]. The design was cross-sectional, all features came from a single automated pipeline, and cognitive impairment was not stratified by etiology. The five algorithms were compared without formal pairwise testing of their AUCs, so Random Forest was selected on numerical performance rather than a statistically significant advantage. The internal estimates are likely optimistic because SMOTE preceded the data split and because both the algorithm and the number of features were chosen using the hold-out set. By contrast, the external validation (AUC 0.860) involved no oversampling or selection and is therefore the more reliable estimate of generalizability. The model’s probability outputs were not calibrated, and we did not quantify the incremental value of multimodal fusion. Future work will confine oversampling and feature selection to the training folds and add a modality ablation, a formal calibration analysis, and a formal comparison of classifier AUCs (e.g., DeLong tests).

## 5. Conclusions

We developed an interpretable multimodal machine-learning model that classifies a MoCA-defined cognitive impairment screening outcome from routine MRI and simple clinical variables. The optimized Random Forest reached a cross-validated AUC of 0.883 internally and 0.860 in independent external validation. In the fitted model, the left MTA score was the leading feature, followed by near-ventricular (juxtaventricular and periventricular) WMH burden and the right MTA score, though the high intercorrelation among WMH features precludes a definitive ranking of individual regions. Relying only on routine sequences and offering SHAP-based interpretability, the model may serve as a screening aid, but it requires prospective multicenter validation and integration with non-imaging biomarkers before clinical use.

## Figures and Tables

**Figure 1:**
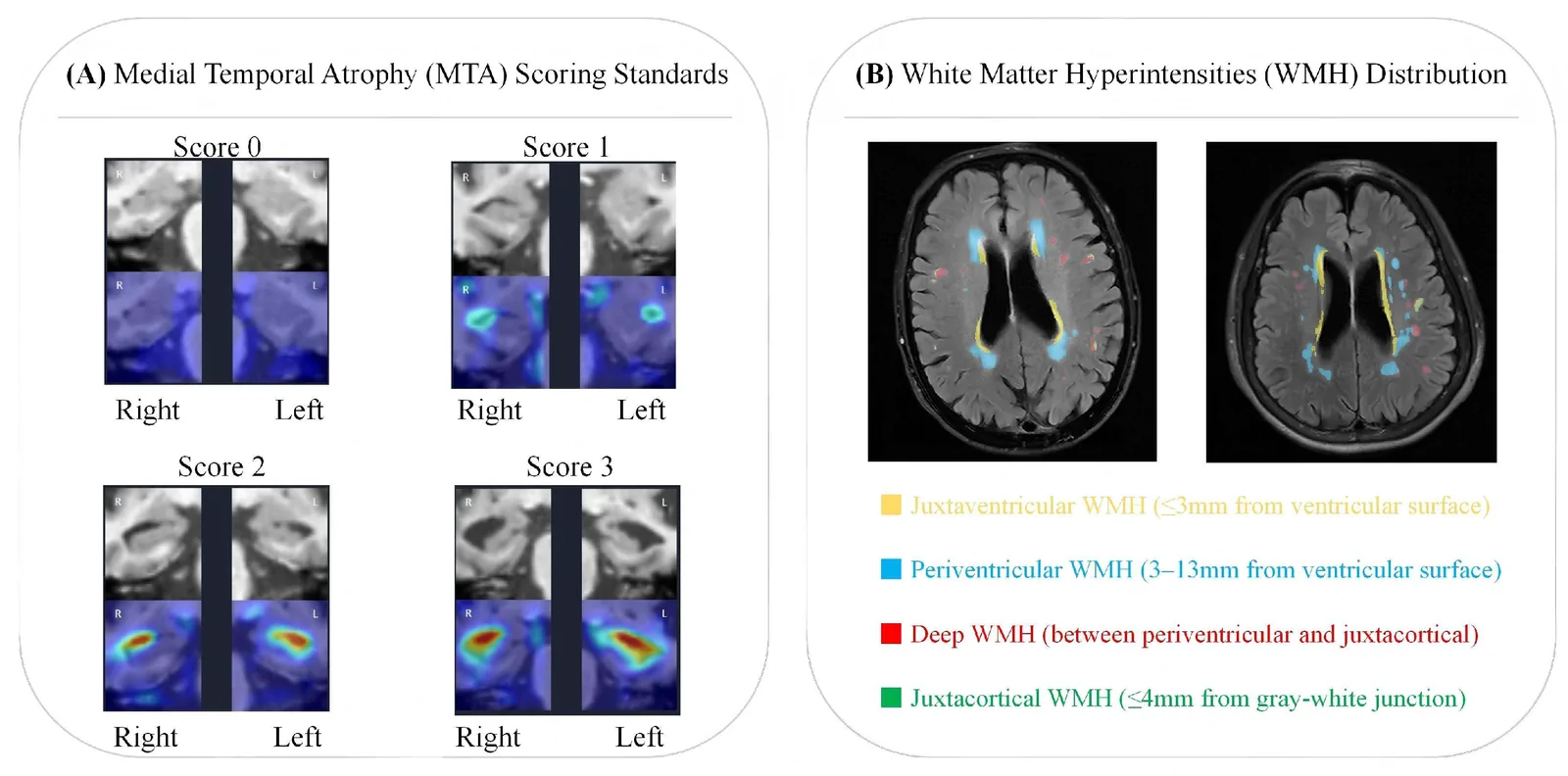
Quantitative MR imaging feature extraction using an AI-based image analysis tool. (**A**) MTA score, which quantifies the degree of atrophy in the hippocampus and surrounding medial temporal lobe structures; scores range from 0 to 4, representing increasing severity of medial temporal lobe atrophy. (**B**) Volume and proportion of WMH in different regions, including juxtaventricular WMH (≤3 mm from the ventricular surface), periventricular WMH (3–13 mm from the ventricular surface), deep WMH (between the periventricular and juxtacortical regions), and juxtacortical WMH (≤4 mm from the gray–white matter junction). MTA, medial temporal atrophy; WMH, white matter hyperintensity.

**Figure 2:**
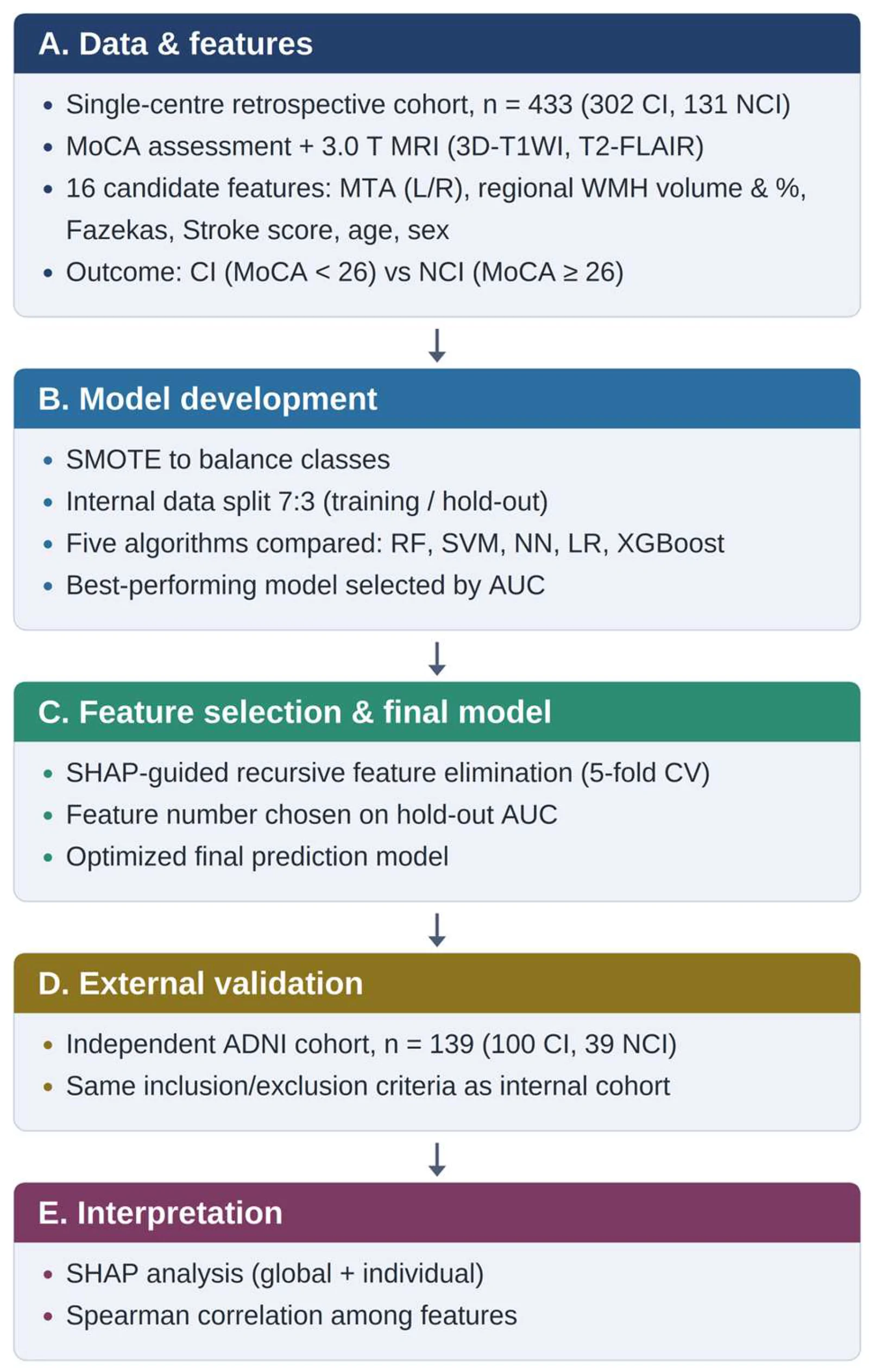
Study workflow. (**A**) A single-center retrospective cohort (*n* = 433; 302 CI, 131 NCI) underwent MoCA assessment and 3.0-T MRI, from which 16 candidate features were extracted. (**B**) SMOTE was applied to the internal dataset before the 7:3 train/hold-out split; models were fitted on the training set, and five algorithms (RF, SVM, NN, LR, XGBoost) were compared on the hold-out set, with the best model selected by AUC. (**C**) SHAP-guided recursive feature elimination was performed with five-fold cross-validation, and the number of features was chosen on the hold-out AUC to obtain the optimized model. (**D**) The optimized model was validated in an independent ADNI cohort (n = 139). (**E**) SHAP analysis and a feature correlation matrix were used for interpretation. Illustrative graphics are schematic and do not represent study results. CI, cognitive impairment; NCI, no cognitive impairment; MoCA, Montreal Cognitive Assessment; MTA, medial temporal atrophy; WMH, white matter hyperintensity; SMOTE, synthetic minority oversampling technique; RF, Random Forest; SVM, support vector machine; NN, neural network; LR, logistic regression; XGBoost, extreme gradient boosting; AUC, area under the curve; ADNI, Alzheimer’s Disease Neuroimaging Initiative; SHAP, SHapley Additive exPlanations.

**Figure 3:**
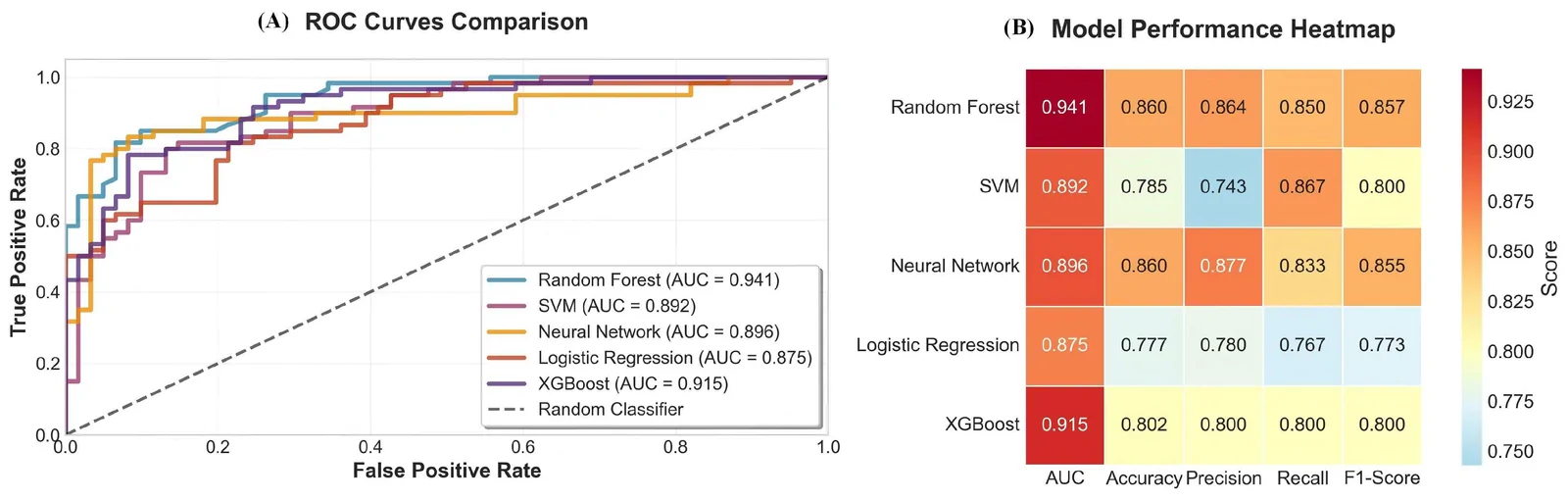
Performance comparison of five machine learning algorithms. (**A**) ROC curves and AUC for all five models. (**B**) Heatmap visualization of all performance metrics across the five algorithms.

**Figure 4:**
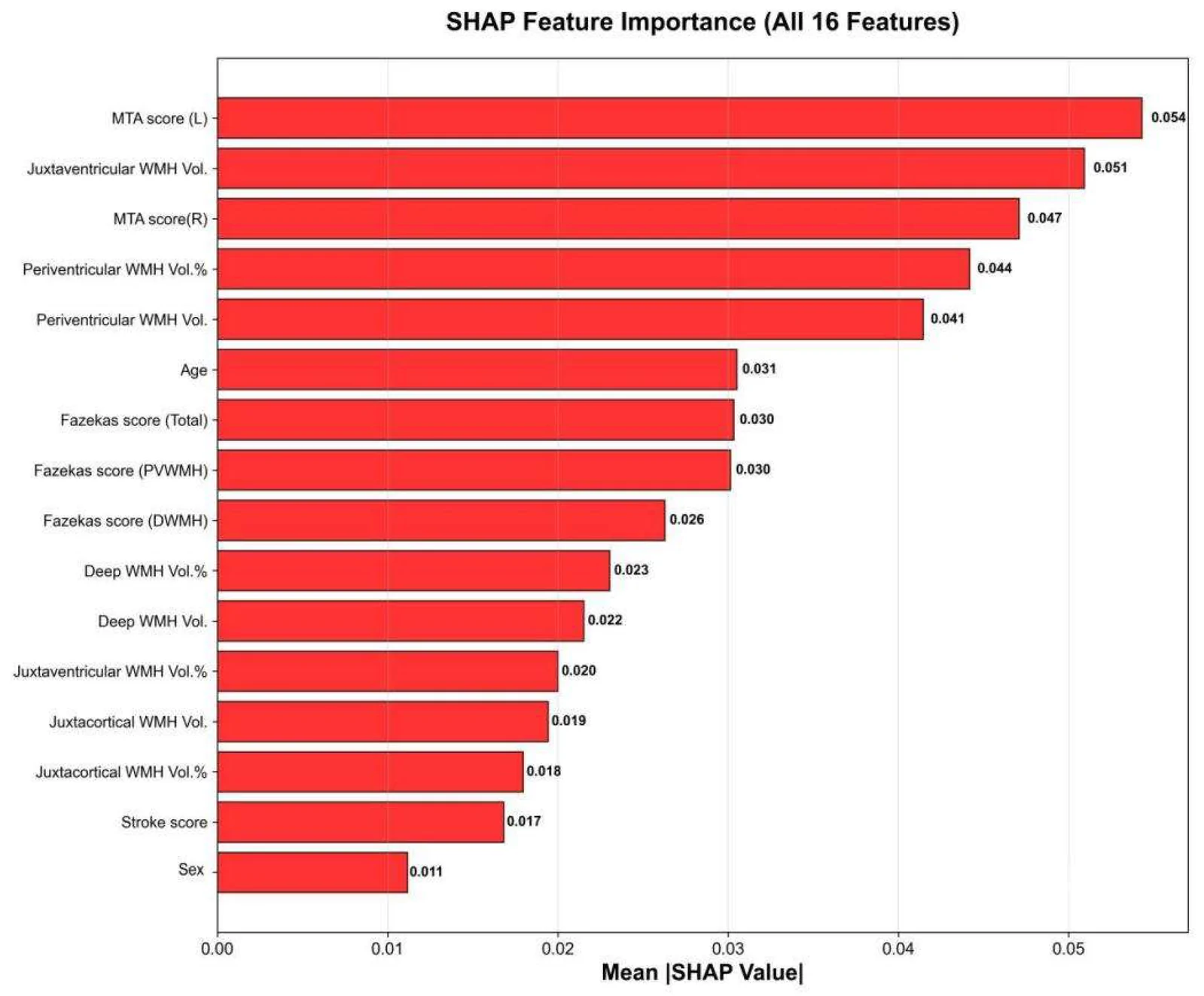
SHAP feature importance analysis for all 16 features. Ranking of the mean absolute SHAP value for each feature, with higher values indicating greater predictive contribution. MTA, medial temporal atrophy; WMH, white matter hyperintensity; PVWMH, periventricular WMH; DWMH, deep WMH; Vol., volume; Vol.%, proportion of WMH volume.

**Figure 5:**
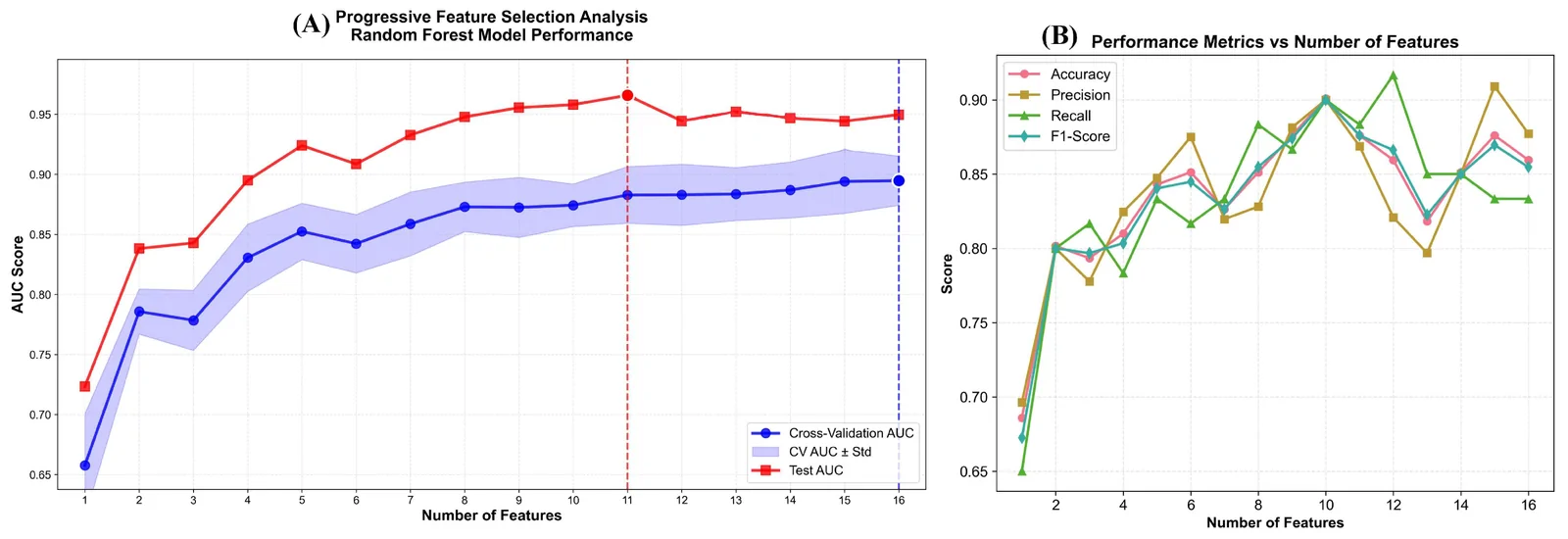
Progressive feature selection analysis for the RF model. (**A**) AUC and cross-validation AUC across models with 1–16 features. (**B**) Four performance metrics (accuracy, precision, recall, and F1 score) for models with different numbers of features. The shaded band around the cross-validated AUC denotes ±1 SD across folds. The cross-validated AUC was stable across the upper part of the path, and the 11-feature model, which gave the highest hold-out AUC, was retained (see Supplementary Table S1).

**Figure 6:**
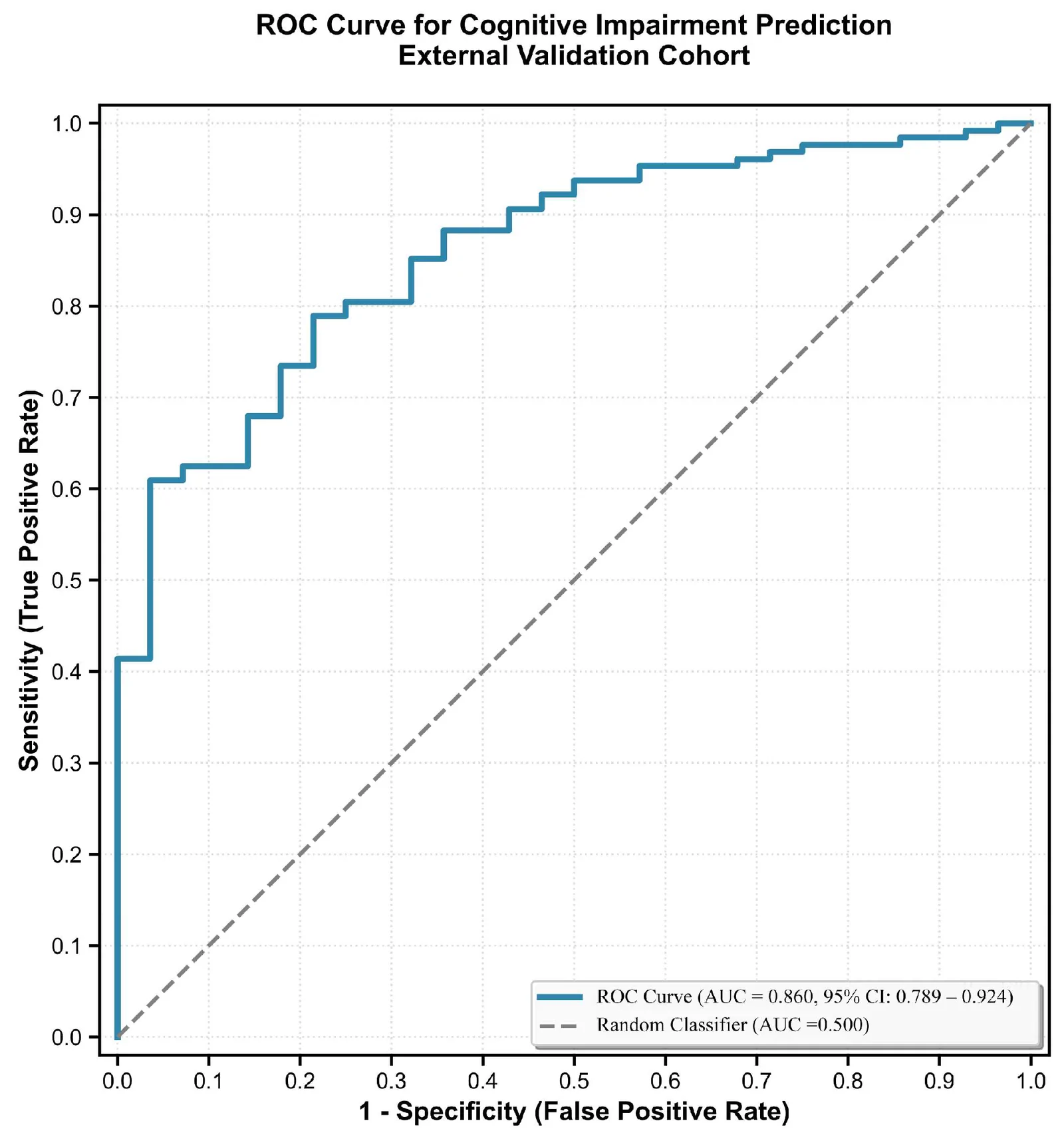
External validation performance of the optimized RF model. ROC curve and AUC of the optimized 11-feature RF model evaluated in the independent ADNI cohort (n = 139). The model achieved an AUC of 0.860 (95% confidence interval, 0.789–0.924).

**Figure 7:**
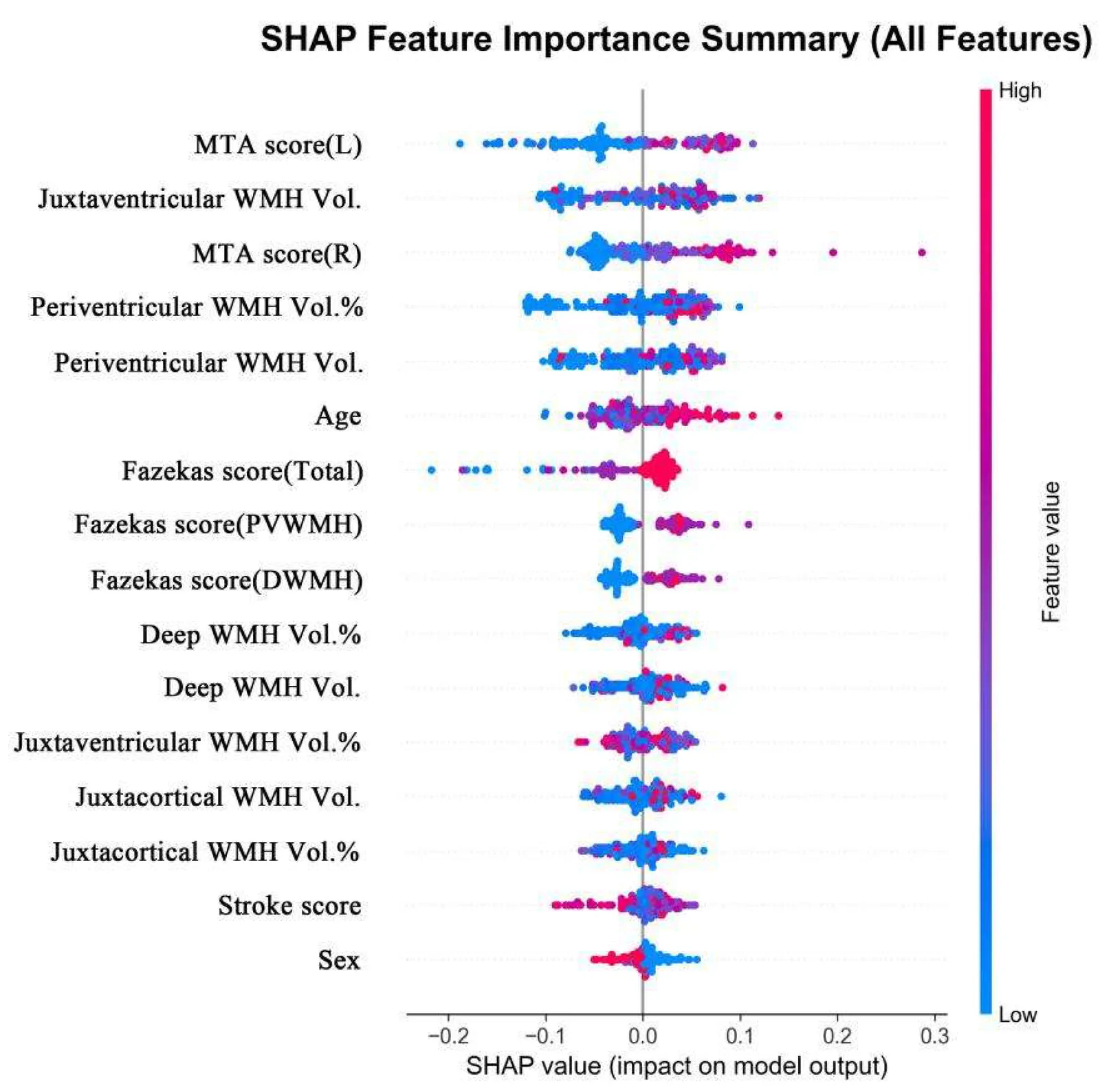
SHAP analysis for the optimized RF model. Beeswarm plot displaying individual feature contributions to predictions, ranked by importance from top to bottom. Color coding represents feature values (red, high; blue, low), and the horizontal position indicates the SHAP value magnitude and direction of impact on the prediction of cognitive impairment. SHAP values refer to the model’s predicted probability of cognitive impairment; positive values indicate a shift toward CI. MTA, medial temporal atrophy; PVWMH, periventricular WMH; DWMH, deep WMH; Vol., volume; Vol.%, proportion of WMH volume.

**Figure 8:**
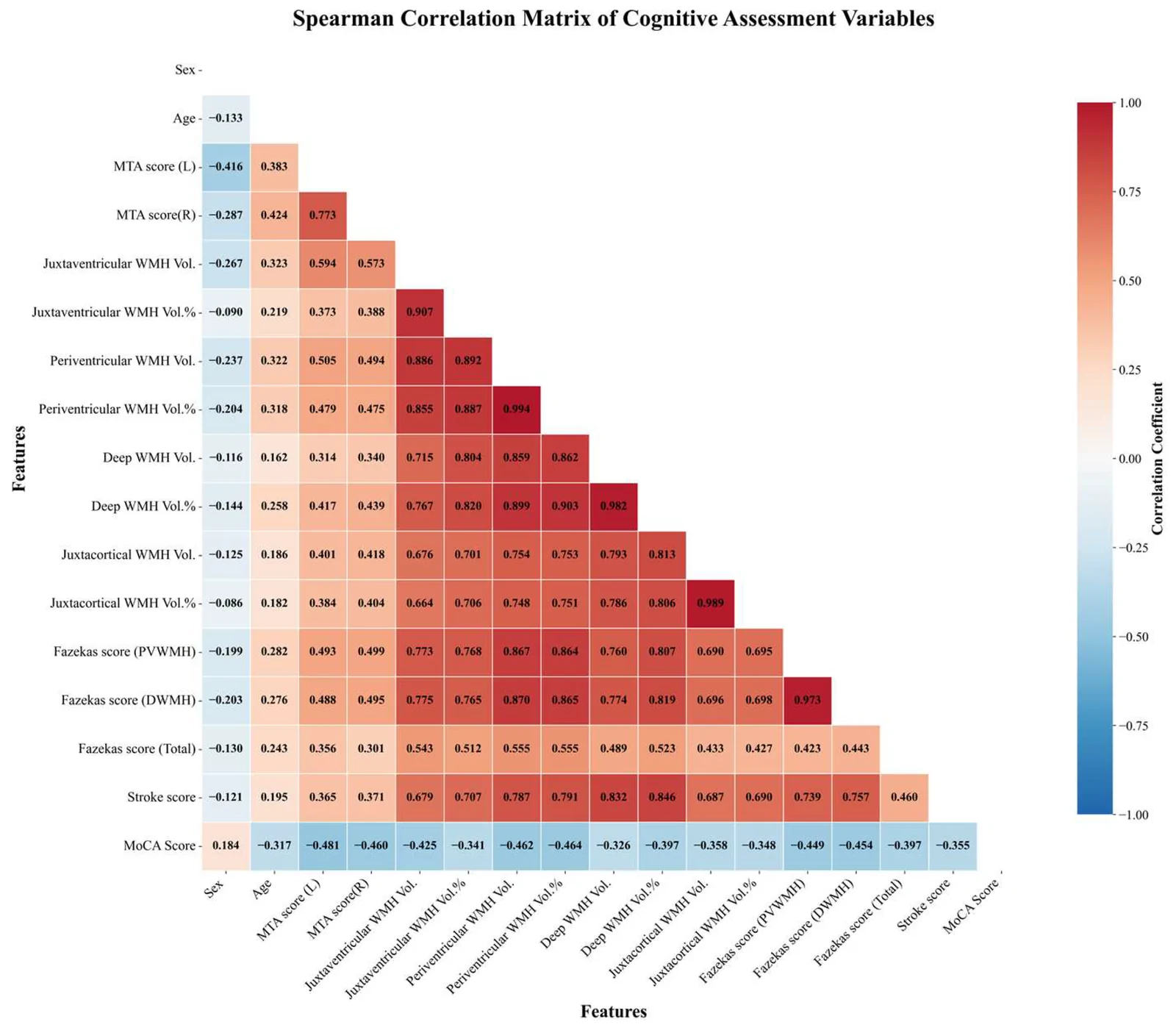
Spearman’s correlation matrix of the assessment variables. Correlation analysis including the selected features and MoCA scores, showing interfeature relationships with correlation coefficients displayed in each cell. The color scale ranges from blue (negative correlation) to red (positive correlation).

**Table 1:** Comparison of clinical characteristics between the cognitive impairment and no cognitive impairment groups.

Group	MoCA	Age (Years)	Sex (F/M)
No cognitive impairment	27 (27–28)	68 (63–72)	86/45
Cognitive impairment	22 (20–24)	72 (67–76)	167/135
Z/χ^2^	−16.601	−5.057	4.02
P	<0.001 *	<0.001 *	0.045 †

* Mann–Whitney U test; † chi-square test. Continuous variables (age, MoCA) are reported as median (IQR) and compared with the Mann–Whitney U test (Z); sex is compared with the chi-square test (χ^2^). P, two-sided.

**Table 2:** Baseline comparison of the internal and external cohorts.

Characteristic	Internal (n = 433)	External/ADNI (n = 139)	P	SMD
Age (years), median (IQR)	70 (65–76)	75 (70–80)	<0.001	−0.55
Female, n (%)	253 (58.4)	56 (40.3)	<0.001	0.37
MoCA, median (IQR)	24 (20–26)	23 (20–26)	0.420	0.09
Cognitive impairment, n (%)	302 (69.7)	100 (71.9)	0.622	−0.05

SMD, standardized mean/proportion difference (|SMD| > 0.10 indicates imbalance). P: Mann–Whitney U (age, MoCA), chi-square (sex, CI prevalence). Distributions of all model predictors are provided in Supplementary Table S2.

## Data Availability

The datasets generated or analyzed during the current study are available from the corresponding author on reasonable request. External validation data are available from the ADNI database (adni.loni.usc.edu).

## Supplementary Materials

The following supporting information can be downloaded at: https://www.mdpi.com/article/10.3390/bioengineering13101107/s1. Table S1: Progressive feature-selection results. Cross-validated AUC (mean ± SD) and hold-out metrics for random-forest models with 1 to 16 features. The 11-feature model, which had the highest hold-out AUC, was retained.; Table S2: Comparison of the model predictors between the internal and external (ADNI) cohorts.; Table S3: External (ADNI) validation performance of the optimized 11-feature random-forest model at the internally fixed operating threshold.; Method Supplement.
