# Supplementary material for: An Interpretable Multimodal Machine-Learning Model for Predicting Cognitive Impairment from Routine MRI: A Study with External Validation
Source: Bioengineering (Basel). 2026 Sep 23;13(10):1107. doi: 10.3390/bioengineering13101107 (PMC13701437; doi:10.3390/bioengineering13101107)
Supplement: Supplementary file 1 [file bioengineering-13-01107-s001.zip › bioengineering-4547283-supplementary.pdf]

Supplementary Materials

Method Supplement

All analyzes were performed in Python 3.8 with scikit-learn 1.0.2, imbalanced-learn 0.9.0, and SHAP 0.41.0. SMOTE used k_neighbors = 5. All random operations used fixed seeds (random_state = 42).

The five algorithms were compared with fixed configurations; no exhaustive hyperparameter search was performed. Explicit settings were as follows. Random Forest used n_estimators = 100 and class_weight = 'balanced'. The support vector machine used a radial-basis-function kernel, probability = True, and class_weight = 'balanced'. The multilayer perceptron used hidden-layer sizes (100, 50) and max_iter = 1000. Logistic regression used class_weight = 'balanced' and max_iter = 1000. XGBoost used eval_metric = 'logloss'. Remaining hyperparameters were left at the library defaults of scikit-learn 1.0.2 or XGBoost and were not tuned. These included C and gamma for the SVM, the activation function, alpha, and initial learning rate for the MLP, the regularization strength C for logistic regression, and n_estimators, learning_rate, max_depth, subsample, and colsample_bytree for XGBoost.

The final 11-feature Random Forest used the same configuration as in the algorithm comparison (n_estimators = 100 and class_weight = 'balanced', with other hyperparameters at library defaults). No additional hyperparameter tuning was applied to the final model.

Table S1. Progressive feature-selection results. Cross-validated AUC (mean ± SD) and hold-out metrics for random-forest models with 1 to 16 features. The 11-feature model, which had the highest hold-out AUC, was retained.

Features	CV AUC	Test AUC	Accuracy	Precision	Recall	F1-Score
16	0.895 ± 0.021	0.950	0.851	0.875	0.817	0.845
15	0.894 ± 0.027	0.944	0.860	0.877	0.833	0.855
14	0.887 ± 0.023	0.947	0.893	0.912	0.867	0.889
13	0.884 ± 0.022	0.952	0.860	0.831	0.900	0.864
12	0.883 ± 0.026	0.945	0.851	0.818	0.900	0.857
11	0.883 ± 0.024	0.966	0.893	0.898	0.883	0.891
10	0.874 ± 0.018	0.958	0.901	0.887	0.917	0.902
9	0.873 ± 0.025	0.956	0.876	0.869	0.883	0.876
8	0.873 ± 0.021	0.948	0.876	0.857	0.900	0.878
7	0.859 ± 0.027	0.933	0.851	0.828	0.883	0.855
6	0.842 ± 0.024	0.909	0.818	0.807	0.833	0.820
5	0.853 ± 0.024	0.924	0.843	0.848	0.833	0.840
4	0.831 ± 0.028	0.895	0.810	0.825	0.783	0.803
3	0.779 ± 0.025	0.843	0.793	0.778	0.817	0.797
2	0.786 ± 0.019	0.838	0.802	0.800	0.800	0.800
1	0.658 ± 0.043	0.723	0.686	0.696	0.650	0.672

Table S2. Comparison of the model predictors between the internal and external (ADNI) cohorts.

Predictor	Internal (n = 433)	External / ADNI (n = 139)	P	SMD
Age (years)	70 (65–76)	75 (70–80)	<0.001	-0.55
MTA score, left	1 (0–1)	1 (1–2)	<0.001	-0.40
MTA score, right	1 (0–2)	1 (1–2)	<0.001	-0.53
Juxtaventricular WMH volume (mL)	3.61 (2.19–5.76)	3.43 (2.10–5.92)	0.905	-0.08
Periventricular WMH volume (mL)	2.28 (0.72–6.11)	1.01 (0.34–3.37)	<0.001	0.21
Periventricular WMH volume (%)	3.62 (1.13–9.12)	1.82 (0.58–5.60)	0.004	0.14
Deep WMH volume (mL)	1.62 (0.60–5.09)	0.06 (0.01–0.24)	<0.001	0.87
Deep WMH volume (%)	1.06 (0.42–3.31)	0.22 (0.05–0.85)	<0.001	0.51
Fazekas score, PVWMH	2 (1–2)	1 (1–2)	<0.001	0.42
Fazekas score, DWMH	2 (1–2)	1 (1–2)	<0.001	0.47
Fazekas score, total	4 (2–4)	2 (2–4)	<0.001	0.45

Values are median (IQR). P, Mann–Whitney U test; SMD, standardized mean difference (|SMD| > 0.10 indicates imbalance; > 0.5 large). MTA and Fazekas are ordinal scores.

Table S3. External (ADNI) validation performance of the optimized 11-feature random-forest model at the internally fixed operating threshold.

Metric	Value
AUC	0.860
Sensitivity	0.870
Specificity	0.513
PPV	0.821
NPV	0.606
F1 score	0.845
TN/FP/FN/TP	20/19/13/87

PPV, positive predictive value; NPV, negative predictive value; TN/FP/FN/TP, true negatives/false positives/false negatives/true positives. Positive class = cognitive impairment (CI). AUC 95% CI: 0.789–0.924.
